# When It Is Better to Regress: Dynamics of Vascular Pruning

**DOI:** 10.1371/journal.pbio.1002148

**Published:** 2015-05-15

**Authors:** Nicolas Ricard, Michael Simons

**Affiliations:** Yale Cardiovascular Research Center, Section of Cardiovascular Medicine, Department of Internal Medicine, Yale University School of Medicine, New Haven, Connecticut, United States of America

## Abstract

Blood vascular networks in vertebrates are essential to tissue survival. Establishment of a fully functional vasculature is complex and requires a number of steps including vasculogenesis and angiogenesis that are followed by differentiation into specialized vascular tissues (i.e., arteries, veins, and lymphatics) and organ-specific differentiation. However, an equally essential step in this process is the pruning of excessive blood vessels. Recent studies have shown that pruning is critical for the effective perfusion of blood into tissues. Despite its significance, vessel pruning is the least understood process in vascular differentiation and development. Two recently published *PLOS Biology* papers provide important new information about cellular dynamics of vascular regression.

## Abstract 2

Blood vessel pruning is an important step in formation and maintenance of effective vascular networks; this Primer assesses two recent papers in PLOS Biology that explore molecular mechanisms of blood vessel regression.

Vascular biology is a rapidly emerging field of research. Given the critical role the vasculature frequently plays in a wide range of common and serious diseases such as arteriosclerosis, ischemic diseases, cancer, and chronic inflammatory diseases, a better understanding of the formation, maintenance, and remodeling of blood vessels is of major importance.

A mature vascular network is a highly anisotropic, hierarchical, and dynamic structure that has evolved to provide optimal oxygen delivery to tissues under a variety of conditions. Whilst much has been learned about early steps in vascular development such as vasculogenesis and angiogenesis, we still know relatively little about how such anatomical and functional organization is achieved. Furthermore, the dynamic nature of mature vascular networks, with its potential for extensive remodeling and a continuing need for stability and maintenance, is even less understood. The issue of optimal vascular density in tissue is of particular importance as several recent studies demonstrated that excessive vascularity may, in fact, reduce effective perfusion [1–3]. Since all neovascularization processes initially result in the formation of excessive amounts of vasculature, be that capillaries, arterioles, or venules, pruning must occur to return the vascular density to its optimal value in order to achieve effective tissue perfusion.

Yet despite its functional importance, little is known about how regression of the once formed vasculature actually happens. While several potential mechanisms have been proposed including apoptosis of endothelial cells, intussusception vascular pruning, and endothelial cell migration away from the regressing vessel, cellular and molecular understanding of how this might happen is conspicuously lacking. Two articles recently published in *PLOS Biology* describe migration of endothelial cells as the key mechanism of apoptosis-independent vascular pruning and place it in a specific biologic context. This important advance offers not only a new understanding of a poorly understood aspect of vascular biology but may also prove to be of considerable importance in the development of pro- and anti-angiogenic therapies.

To put vessel regression in context, it helps to briefly outline the current understanding of vessel formation. During embryonic development, vasculature forms in several distinct steps that begin with vasculogenesis, a step that involves differentiation of stem cells into primitive endothelial cells that then form initial undifferentiated and nonhierarchically organized lumenized vascular structures termed the primary plexus [4]. The primary plexus is then remodeled, by the process termed angiogenesis, into a more mature vascular network [5]. This remodeling event involves both formation of new vessels accomplished either by branching angiogenesis, a process dependent on tip cell-driven formation of new branches [6], or intussusception, a poorly understood process of splitting an existing vessel into two [7]. This incompletely differentiated and still nonhierarchical vasculature then further remodels into a number of distinctly different types of vessels such as capillaries, arteries, and veins. This requires fate specification, differentiation, and incorporation of various mural cells into evolving vascular structures. Finally, additional specialization of the vascular network occur in an organ-specific manner.

Once formed, vascular networks require active maintenance as withdrawal of key signals, such as of ongoing fibroblast growth factor (FGF) or vascular endothelial growth factor (VEGF) stimulation, can lead to a rapid loss of vascular integrity and even changes in endothelial cell fate [8–12]. In addition, mature vessels retain the capacity for extensive remodeling and new growth as can be seen in a number of conditions from cancer to myocardial infarction and wound healing responses, among many others [5].

A key issue common to both embryonic and adult vessel remodeling is how an existing lumenized vessel connected to the rest of the vasculature undergoes a change that results in its remodeling into something else. Such a change may involve either a new branch formation or regression of an existing branch, while the patency and integrity of the remaining circulation is maintained. Two types of cellular process leading to branching have been described—sprouting and intussusception. Formation of vascular branches by sprouting involves VEGF-A-induced expression of high levels of delta-like ligand 4 (Dll4) in a subset of endothelial cells at the leading edge of the vascular sprouts that are lying closest to the source of VEGF, thus converting them to a “tip cell” phenotype. Some of the key features of tip cells include the presence of cytoplasmic processes that extend into avascular (or hypoxic) tissue that form nascent branches. Dll4 expressed on tip cells binds Notch-1 receptor in neighboring endothelial cells, thereby activating their downstream Notch signaling. In turn, Notch signaling shuts down the formation of additional filopodia processes, converting these cells to a “stalk cell” phenotype and thereby avoiding excessive branching [13–15]. The bone morphogenetic protein signaling pathway provides further input in determining stalk cell fate [16]. Importantly, tip cells are only partially lumenized; only once they have converted to a stalk phenotype does the lumen extend to what was a tip cell and its sprouts.

An alternative mechanism of branching involves intussusception, a process by which a tissue pillar from the surrounding tissue splits the existing endothelial tube into two along its long axis, creating two adjusting vessels. While this process has been described morphologically, virtually nothing is known about its molecular and cellular regulation. In development, angiogenesis by intussusception occurs in vessels previously formed by sprouting angiogenesis [17,18]. Importantly, however, both sprouting angiogenesis and intussusception allow growth and remodeling of vascular network without any integrity compromise, thereby avoiding bleeding and related complications.

There are certain parallels between vessel formation and branching and vessel regression. While growth occurs either via sprouting (a process linked to endothelial cell-migration) or intussusception, regression involves either “reverse intussusception,” endothelial migration-dependent regression, or apoptosis. The latter is the primary means of regression of the hyaloid vasculature in the eye and of the vascular loss seen in oxygen-induced retinopathy (OIR). In the case of hyaloid vasculature, secretion of WNT7b by macrophages invading the hyaloid membrane induces apoptosis of hyaloid endothelial cells leading to the regression of the entire hyaloid vasculature [19]. This total apoptosis-induced loss of hyaloid blood vessels contrasts with a less extensive vascular regression seen in the setting of OIR. In this condition, exposure of the developing retinal vasculature to abnormally high oxygen levels leads to vascular damage characterized by capillary pruning [20]. The pruning is the consequence of apoptosis of endothelial cells due to the toxic effect of a combination of high oxygen and low VEGF level. Interestingly, larger vessels and mature capillaries are not sensitive to hyperoxia [21].

Intussusception vascular pruning was also described in a low VEGF level context in the chick chorioallantoic membrane. Application of VEGF-releasing hydrogels to the membrane surface results in formation of an excessive vasculature. Removal or degradation of the hydrogel induces an abrupt VEGF withdrawal. In this context, formation of transluminal pillars, similar to the ones seen in intussusception angiogenesis, is observed in vessels undergoing pruning [22]. The same process is observed in the tumor vasculature in the setting of anti-angiogenic therapy [23]. Finally, apoptosis-independent vascular regression, driven by endothelial cell migration, has been described in the mouse retina, yolk vessels of the chick and mouse embryos, branchial arches, and the zebrafish brain [24–28].

In all of these cases, only a subset of vessels is designated for pruning, and the selection of these vessels is highly regulated. Yet, factors involved in choosing a particular vascular branch for pruning remain ill-defined. One such factor is low blood flow [27,28]. Another is Notch signaling that has been shown to at least partially control vascular pruning in mouse retina and in intersegmental vessels (ISVs) in zebrafish [24]. Loss of Notch-regulated ankyrin repeat protein (Nrarp), target gene of Notch signaling, leads to an increase in vascular regression in these tissues due to a decrease in Wnt signaling-induced stalk cell proliferation. Similarly, in *Dll4* ^*+/-*^ mice, developmental retinal vascular regression and OIR-induced vascular pruning are reduced [29], confirming the involvement of the Notch pathway in the control of vascular regression.

The two factors may be linked, as low flow can affect endothelial shear stress and lead to a decrease in Notch activation. Such a link is suggested by studies on vascular regression in mice with endothelial expression of dominant negative NFκB pathway inhibitor that demonstrate excessive vascular growth but reduced tissue perfusion [2]. Molecular studies showed inhibition of flow- or cytokine-induced NFκB activation results in decreased Dll4 expression [2].

Another important issue is the fate of endothelial cells from vessels undergoing pruning. In *PLOS Biology*, two groups recently described endothelial cell behavior during vascular pruning in three different models: the mouse retina, the ISVs in zebrafish, and the subintestinal vessel in zebrafish [30,31]. Using a high resolution time-lapse microscopy technique, Lenard and collaborators showed that vascular pruning during the subintestinal vessel formation occurs in two different ways. In type I pruning, the first step is the collapse of the lumen. Once that occurs, endothelial cells migrate and incorporate into the neighboring vessels. In type II pruning, the lumen is maintained. One endothelial cell in the center of the pruning vessel undergoes self-fusion, leading to a unicellular lumenized vessel. At the same time, other endothelial cells migrate away and incorporate into the neighboring vessels. The eventual lumen collapse is the last step after which the remaining single endothelial cell migrates and incorporates into one of the major vessels.

Franco and collaborators described a pruning mechanism similar to the type I pruning described by Lenard et al., showing lumen disruption as an initial step in pruning of retinal vasculature in mice and ISVs in zebrafish [31]. By analyzing the first axial polarity map of endothelial cells in these models, they demonstrated that axial orientation predicts endothelial cell migration, and that migration-driven pruning occurs in vessels with low flow. Interestingly, migrating endothelial cells in regressing vessel display a tip cell phenotype with filopodia.

The cellular dynamic of vessel pruning described here is the reverse of the cellular dynamic during anastomosis and angiogenesis [32]. Given the crucial role of factors as VEGF for the migration of endothelial cells during angiogenesis, can we go further and propose that other cytokines or cell–cell signaling may be involved in the migration of these endothelial cells? Indeed, low blood flow seems to be the cause of vessel pruning, but how can we explain the direction of endothelial cell migration, moreover with a tip cell morphology? Also, what determines the choice between type I and type II pruning? The collapse of lumen suggests a reorganization of the cytoskeleton, and a loss of polarity and electrostatic repulsion of endothelial cells. Molecular mechanisms leading from low shear stress to loss of endothelial cell polarity need further investigation. As defective vascular pruning could be involved in poor recovery after injury or ischemic accident, a better understanding of the molecular control of this phenomenon appears to have medical consequences. Another question that is still unanswered is the fate of mural cells that surrounded the pruned vessels. Small vessels are covered by pericytes, which have strong interaction with endothelial cells. How and when are these interactions disrupted? Are pericytes integrated into the neighboring vessel, or do they undergo apoptosis? Further studies are needed to understand the molecular and cellular mechanisms by which vasculature can adapt, even at the adult stage, to support the nutrient and oxygen needs of each cell.

Overall, taking the results of these studies together with other recent developments in this field, the following picture is emerging (Fig 1). Under conditions of low blood flow in certain vascular tree branches, pruning will occur via endothelial cell migration out of these branches to the neighboring (presumably higher blood flow) vessels. This results in decreased total vascular cross-sectional area and increased average blood flow, thereby terminating further pruning. Importantly, this occurs without the loss of luminal integrity and without reduction in the total endothelial cell mass. At the same time, vessels that suddenly find themselves in a low VEGF environment will regress either by apoptosis of endothelial cells or by intussusception. In both cases, there is a reduction in the total vasculature without an increase in blood flow to this tissue. Thus, the local context determines the mechanism: migratory regression and remodeling in low shear stress versus apoptotic pruning in low VEGF milieu.

**Fig 1:**
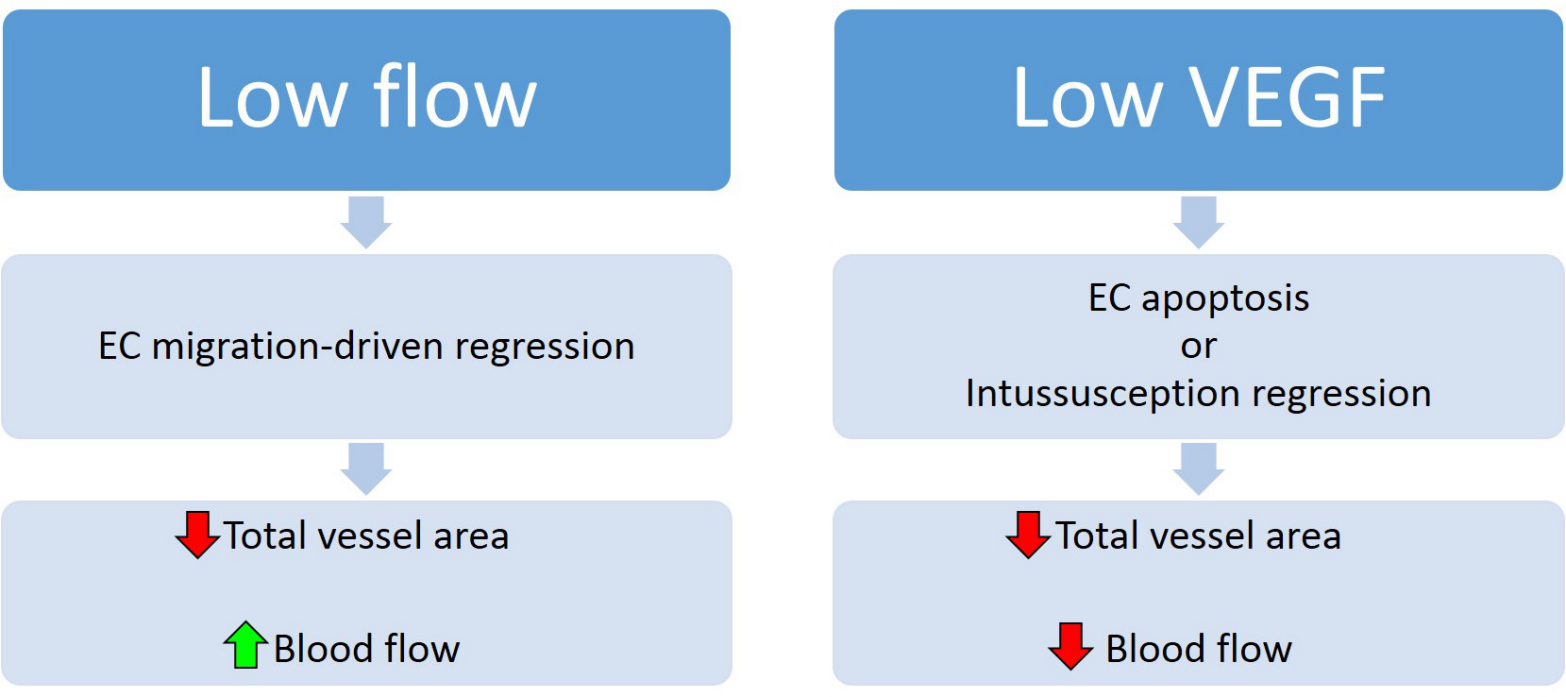
Vessel regression under low flow versus low VEGF conditions. Vessel regression under low flow conditions proceeds by endothelial cell (EC) migration-driven regression, resulting in a decrease in total vessel areas but an increase in blood flow (left panel). Vessel regression under low VEGF conditions proceeds by EC apoptosis or intussusception regression, resulting in decreased vessel number and decreased flow to tissues subtended by the regressing vasculature (right panel). Image credit: Nicolas Ricard & Michael Simons.

This distinction is likely to be of a significant practical importance, in particular in the context of therapies designed to facilitate vessel normalization in tumors after VEGF-targeting treatments and therapies designed to promote vascularization of mildly ischemic tissues as occurs, for example, in the setting of chronic stable angina and other similar conditions. In the former case, a precipitous drop in VEGF levels is likely to induce vascular regression by induction of endothelial apoptosis, and further promotion of apoptosis may facilitate this process. In contrast, in the latter case, low flow in newly formed collateral arteries may induce their regression by stimulating outmigration of endothelial cells, thereby limiting their beneficial functional impact. Therapies designed to inhibit this mechanism, therefore, may promote growth of the new functional vasculature.

## Abbreviations

Dll4: delta-like ligand 4
FGF: fibroblast growth factor
ISV: intersegmental vessel
Nrarp: Notch-regulated ankyrin repeat protein
OIR: oxygen-induced retinopathy
VEGF: vascular endothelial growth factor
